# Relationship between shoulder and elbow range of motion and ultrasonographic structural abnormalities in the elbow of Taiwanese high school baseball players

**DOI:** 10.1186/s13102-024-00839-z

**Published:** 2024-02-12

**Authors:** Yi Lu, Poyu Chen, Wen-Yi Chou, Cheng-Pang Yang, Huan Sheu, Hao-Che Tang, Chun-Jui Weng, Joe Chih-Hao Chiu

**Affiliations:** 1 Department of Orthopedic Surgery, Linkou Chang Gung Memorial Hospital, Taoyuan, Taiwan; 2 Bone and Joint Research Center, Linkou Chang Gung Memorial Hospital, Taoyuan, Taiwan; 3 Comprehensive Sports Medicine Center (CSMC), Chang Gung Memorial Hospital, Taoyuan, Taiwan; 4 Department of Occupational Therapy and Graduate Institute of Behavioral Sciences, College of Medicine, Chang Gung University, Taoyuan, Taiwan; 5 Department of Orthopedic Surgery, Kaohsiung Chang Gung Memorial Hospital, Chang Gung University College of Medicine, Kaohsiung, Taiwan; 6 Department of Orthopedic Surgery, Taoyuan Chang Gung Memorial Hospital, Taoyuan, Taiwan; 7 Department of Orthopedic Surgery, Keelung Chang Gung Memorial Hospital, Keelung, Taiwan; 8 Department of Orthopedic Surgery, Kaohsiung Municipal Feng-Shan Hospital, Kaohsiung, Taiwan; 9 School of Medicine, Chang Gung University, No. 5, Fusing St., Gueishan District, Taoyuan City, 333 Taiwan

**Keywords:** Baseball, Elbow, Shoulder, Range of motion, Ulnar collateral ligament, ultrasound

## Abstract

**Background:**

Ultrasonographic structural abnormalities are regarded as one of the risk factors of elbow injuries. Elbow injuries are commonly associated with decreased shoulder/elbow range of motion (ROM). The purpose of this study is to determine the relationship between shoulder/elbow ROM and elbow ultrasonographic structural abnormalities in Taiwan high school baseball players.

**Methods:**

A total of 533 Taiwan high school baseball players were enrolled. Physical examinations including measurements on shoulder/elbow ROM and elbow sonographic examinations were performed and recorded by professional physicians. The analyses were conducted in three subgroups according to their defensive position because the training programs were different. All players pooled, pitchers-only, and fielders-only, due to several demographic differences among these subgroups. In all the subgroups, univariate analyses were conducted separately for participants with and those without elbow ultrasonographic structural abnormalities, and then multivariate analyses were conducted to identify factors significantly related. The odds ratios (ORs) were used to estimate the risk of elbow ultrasonographic structural abnormalities.

**Results:**

Demographic data showed that pitchers had taller body height (*P* < 0.001) and greater elbow flexion/extension ROM (*P* < 0.001). When all players were pooled, significant risk factors included started playing baseball at an younger age (OR = 1.202; 95% CI = 1.064–1.357; *P* = 0.003), longer experience of official baseball (OR = 1.154; 95% CI = 1.038–1.283; *P* = 0.008), lower total shoulder rotation angle (OR = 1.007; 95% CI = 1.000–1.014; *P* = 0.050), and less total elbow arm angle (OR = 1.052; 95% CI = 1.017–1.088; *P* = 0.003) For pitchers, significant risk factors included longer experience of official baseball (OR = 1.342; 95% CI = 1.098–1.640; *P* = 0.004), lower total shoulder rotation angle (OR = 1.016; 95% CI = 1.004–1.027; *P* = 0.006), and lower total elbow arm angle (OR = 1.075; 95% CI = 1.024–1.129; *P* = 0.004) (Table 5). There were no significant risk factors for elbow structural abnormalities in fielders.

**Conclusion:**

For Taiwan high school pitchers, longer official baseball experience, decreased shoulder total rotational angle, and decreased elbow total flexion/extension angle, were related to ultrasonographic structural abnormalities in elbows.

## Highlights

**What is known about this subject**

Several studies address the relationship between shoulder/ elbow range of motion and elbow injuries in baseball players.

Clinical presentations such as decreased pitch velocity and accuracy, local tenderness, pain during throwing, are related to elbow injuries at late stage.

Structural abnormalities could serve as “pre-injury” status of youth baseball player.

**What this study adds to existing knowledge**

In youth baseball players, decreased shoulder and elbow range of motion were significantly related to elbow structural abnormalities, especially for pitchers.

This could provide not only physicians, but also coaches, trainers, and the players a simple way to examine their “pre-injury” status.

Longer official baseball experience could increase elbow structural abnormalities.

## Introduction

Elbow injuries are common among young baseball players [1, 2]. Elbow injuries in youth not only delay the players’ training schedules, technique developments, and decrease game participation, but also increase the risk of future injuries [3]. In the past 10 years, approximately 5% of young baseball pitchers in the United States required surgery or retirement from baseball because of elbow injuries [4]. As a result, evaluating the potential risk factors for elbow injuries in young baseball players is of great importance. According to Sakata et al., the return-to-play (RTP) rate among high school baseball players with ulnar collateral ligament (UCL) injuries managed with nonoperative treatments was as high as 83.6% [5]. The high RTP rate was because the injury was mostly type I or type II, indicating partial tear over UCL [6]. Aside from UCL injuries, elbow tendinitis, tenosynovitis, epicondylitis and spirochetes were also prominent in adolescent pitchers, which could also be presented with structural abnormalities [7]. 

Hence, it is important for physicians, coaches, and trainers to detect injuries among young players in an early stage and modify the training protocol accordingly.

Clinical presentations for elbow injuries encompass local pain or tenderness, loss of throwing velocity and accuracy, and subjective feeling of instability [8, 9]. However these symptoms are usually present when the injuries are already severe, such as, complete ligamentous tear or high-grade partial tear of elbow UCL [10]. As a consequence, several studies aimed to detect injuries in an early stage with different ways, including frequent physical examinations, ultrasonographic studies, and analysis on throwing mechanism [11–13]. In a meta-analysis conducted by Pozzi et al., pre-season screening of shoulder external rotation range of motion (ROM) can identify professional baseball pitchers who are at risk of elbow injury [14]. In a clinical study conducted by Harada et al., shoulder and elbow structural abnormalities detected by ultrasonography were significantly related to injuries in the future [15]. 

In addition, several environmental-specific factors (such as pitchers when compared to being position players) and individual-specific factors (such as pitching velocities) have been regarded as risks for elbow injuries in several studies [1, 16]. For these players assessing the risks, early detection of injuries and close observation during games and training are more important.

In this study, we analyzed factors related to structural abnormalities in the elbows of young baseball players. We utilized ultrasonography and a thorough physical examination as screening tools, as they can be performed more easily and quickly than other examinations such as magnetic resonance imaging (MRI) or computer tomography. These factors could be meaningful not only for physicians and surgeons but also for coaches, physical therapists, and players to detect elbow injuries earlier.

We believe that detecting elbow structural abnormalities in youth baseball players is crucial, as these abnormalities could impact players’ performance and may also serve as indicators of pre-injury status [17]. As a result, this study aimed to (1) determine the risk factors for elbow structural abnormalities and (2) identify the relationship between elbow structural abnormalities and ROM of the shoulder and elbow via physical and ultrasonographic examinations among Taiwanese high school baseball players. We hypothesized that shoulder and elbow ROM may be related to ultrasonographic structural abnormalities in Taiwanese high school players.

## Methods

### Study design

This is a retrospective, cross-sectional study.

### Players

In the period of 2016–2017 Taiwan high school baseball season, comprehensive physical and ultrasonographic examinations were performed prospectively on players in 15 elite baseball high schools. Inclusion criteria were willingness to participate in this program, absence of any surgical history of the shoulder or elbow, and the ability to fully participate in all baseball-related activities without physical or mental restrictions. Players were excluded if they were unable to fully participate in baseball activities for any reason, had a prior injury from which they had not fully recovered, or were unwilling to participate in the physical and ultrasonographic examinations. Player demographics, such as age, number of years played, height, weight, body mass index (BMI), dominant throwing side, and primary defensive positions, were recorded. This study was approved by the institutional review board of the authors’ institution (IRB 202101106A3D001).

### Assessment of motion

**Fig. 1:**
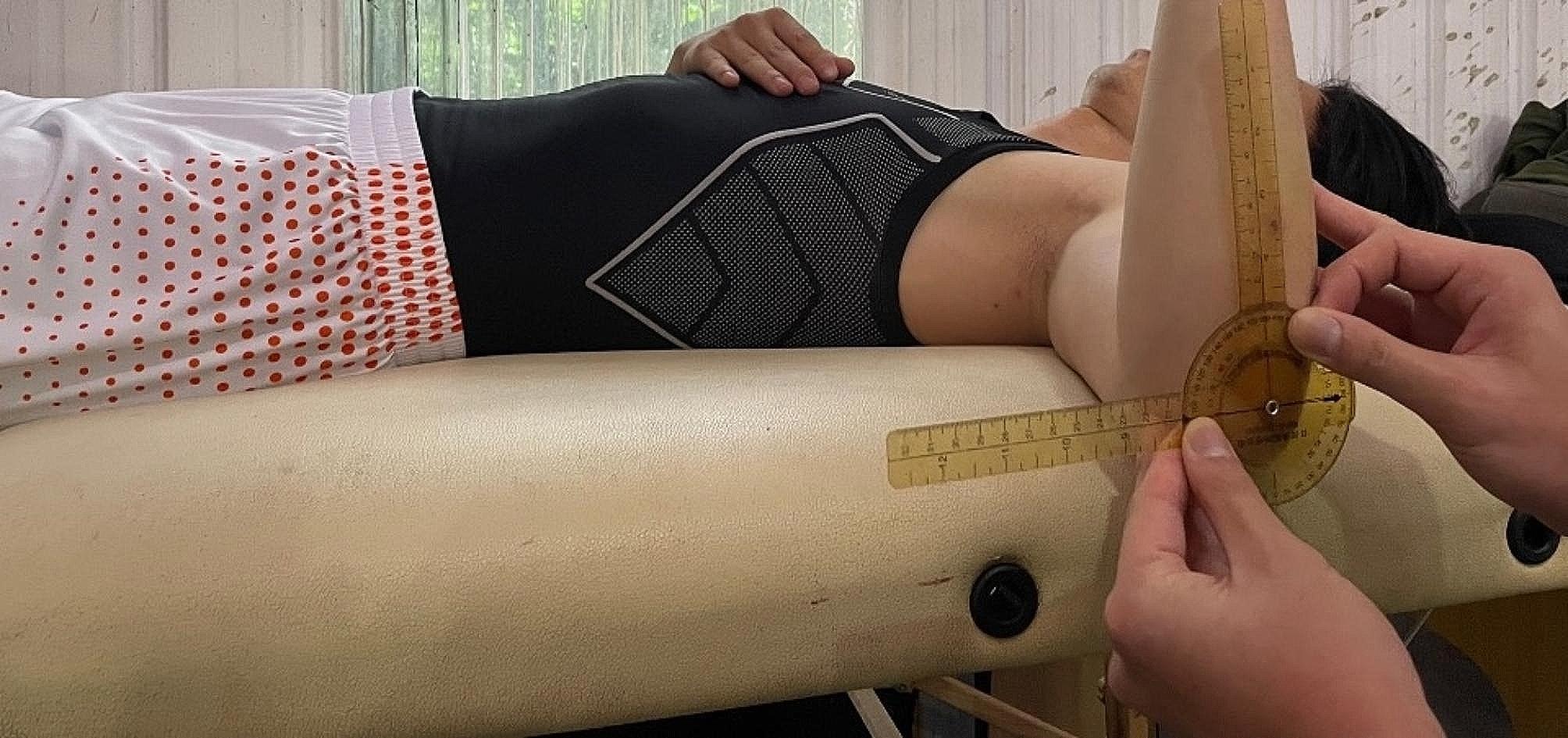
The patient was set in supine position. Neutral position of external and internal rotation of shoulder was set upon anterior arm perpendicular to the plane

**Fig. 2:**
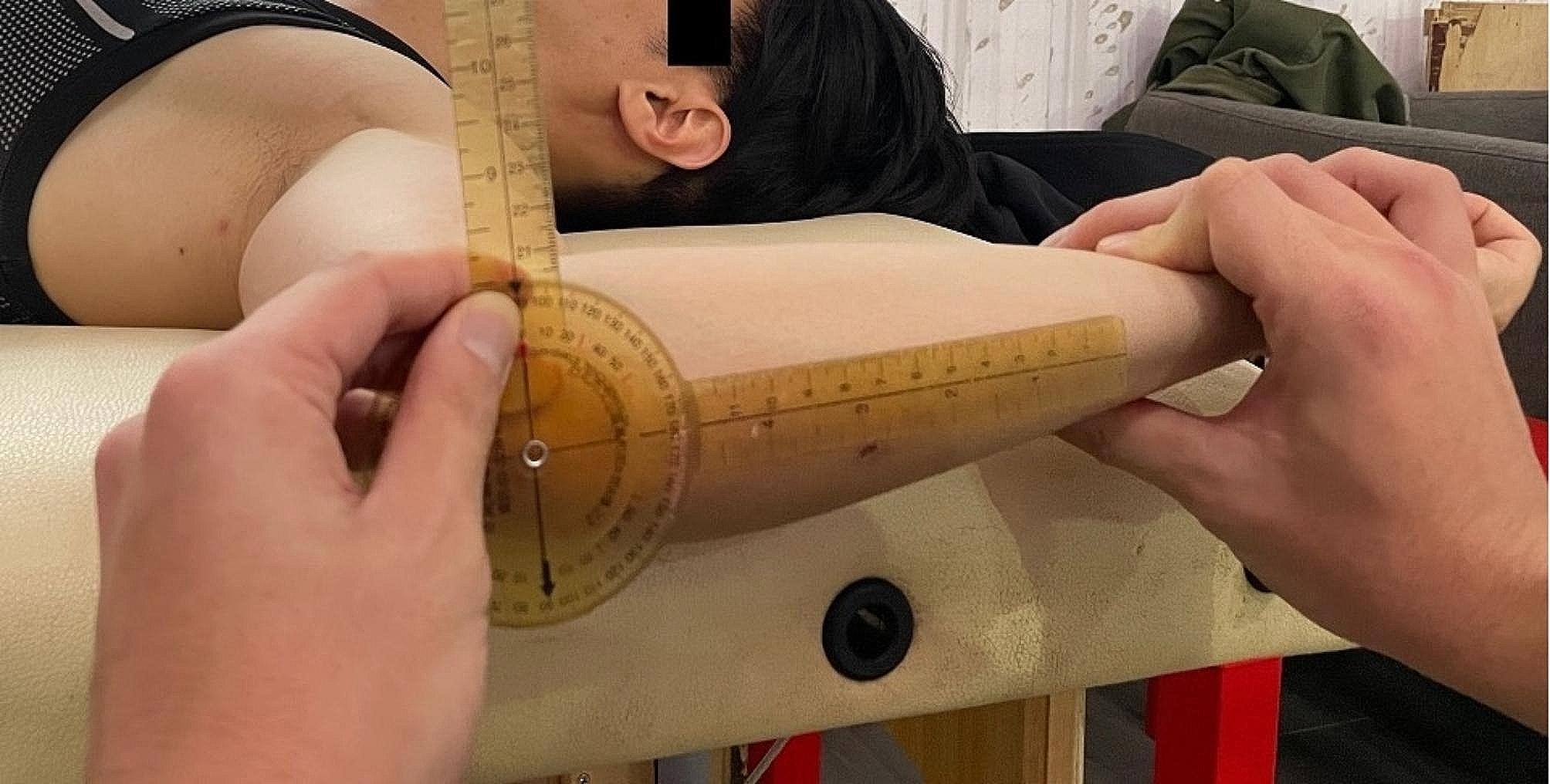
External rotational angle of shoulder was the angle measured between full passive external rotation and the neutral position

**Fig. 3:**
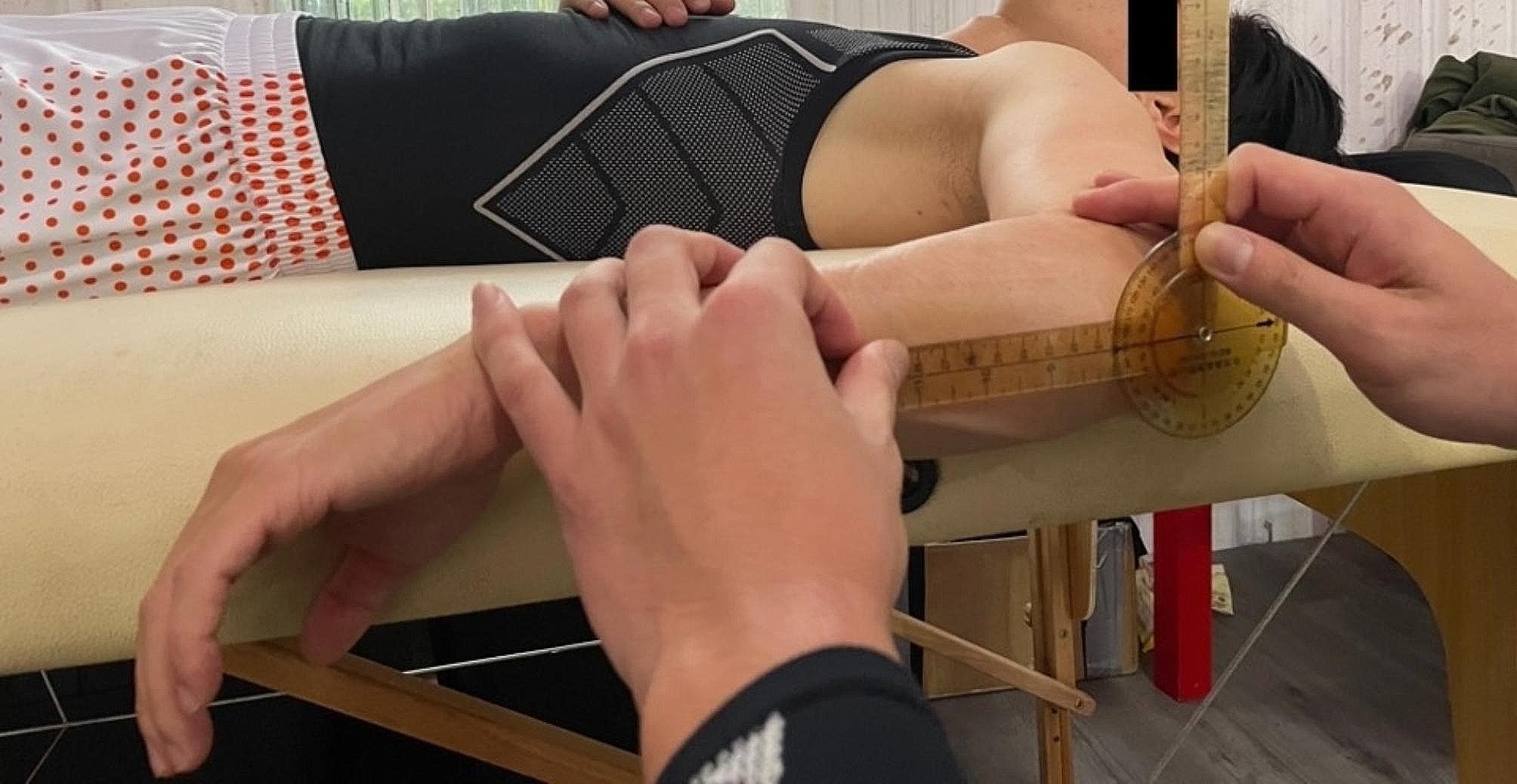
Internal rotational angle of shoulder was the angle measured between full passive internal rotation and the neutral position

Shoulder ROM was assessed in the supine position, as described by Wilk et al. (Figs. 1, 2 and 3). Shoulder external rotation (ER) and internal rotation (IR) were measured with the patient in a supine position and the arm was in 90° of abduction.

The stationary arm of the goniometer was placed along a line perpendicular to the table and the axis of rotation was through the olecranon. The moving arm of the goniometer was placed along the posterior ulnar border and degrees of motion were recorded based on the movement of the ulna [18]. Elbow ROM was assessed with the players seated.

**Fig. 4:**
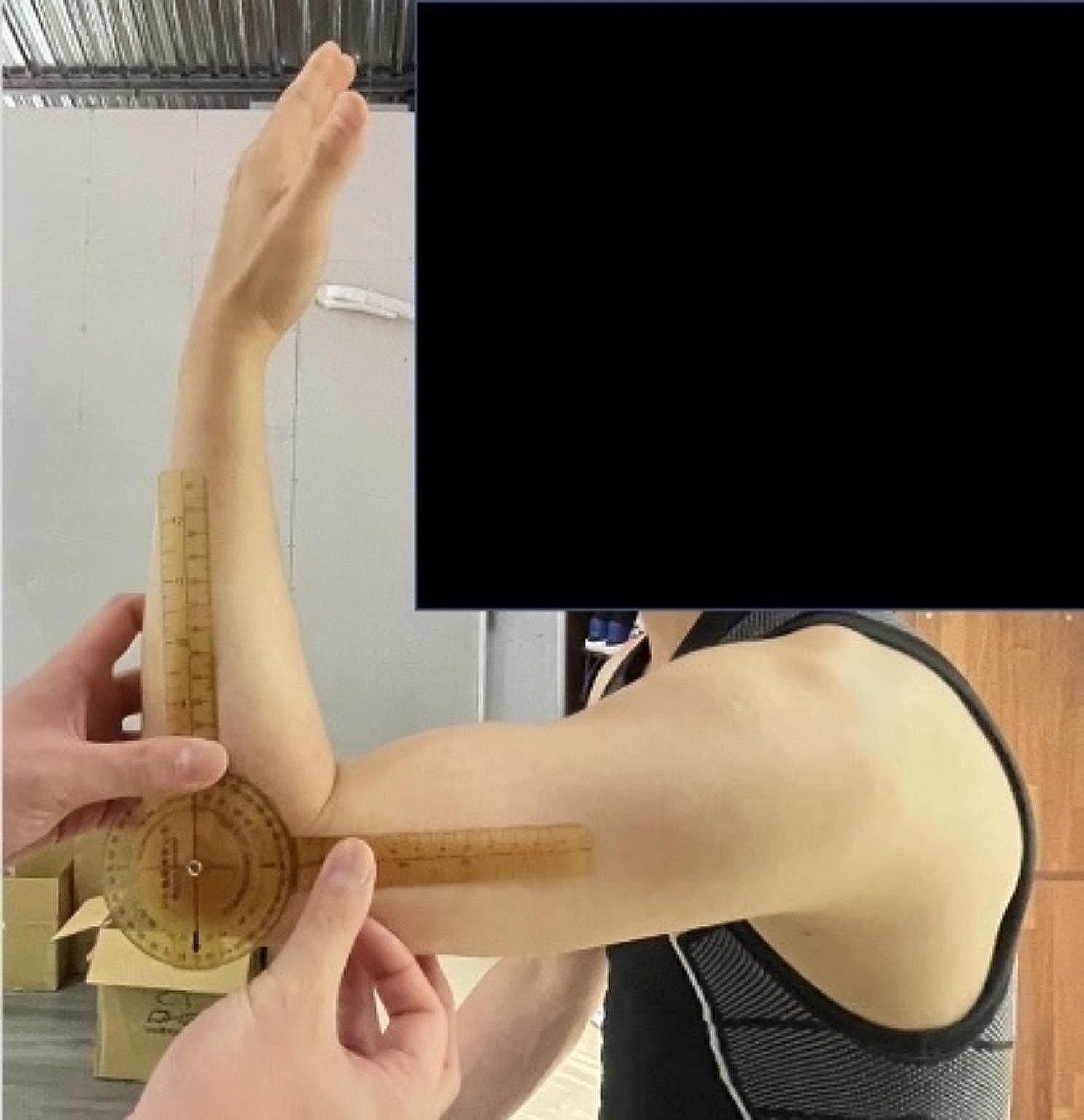
The patient was seated, with his upper arm parallel to the ceiling. Neutral position of flexion and extension of elbow was set upon anterior arm perpendicular to upper arm

**Fig. 5:**
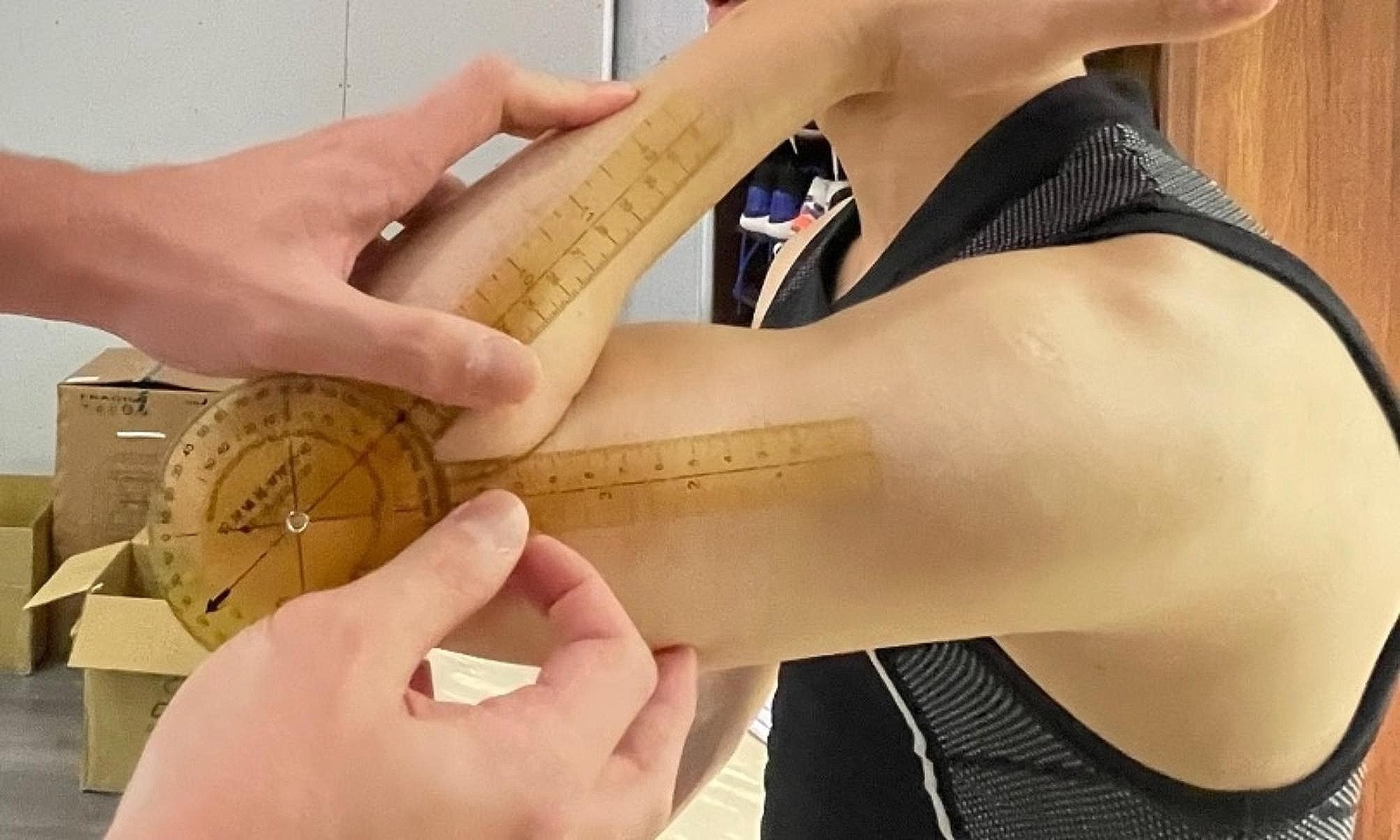
Flexion angle of elbow was the angle measured between full passive flexion and the neutral position

**Fig. 6:**
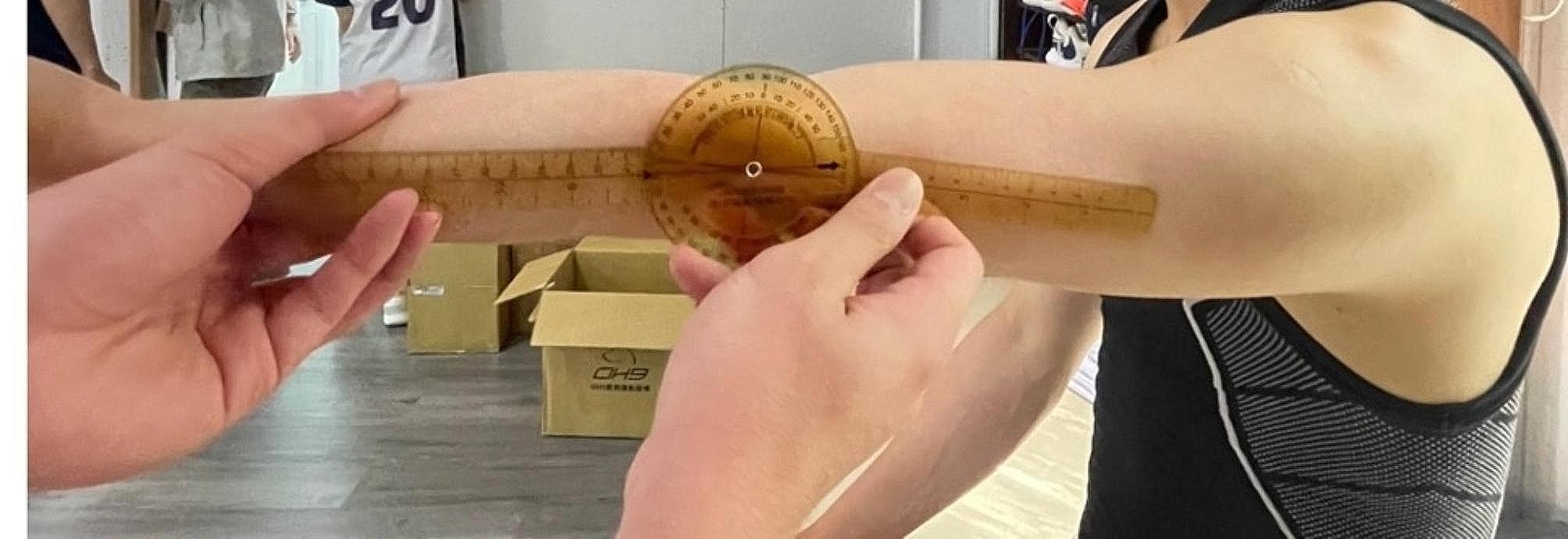
Extension angle of elbow was the angle measured between full passive extension and the neutral position

The elbow total arm angle was assessed with the player seated, the arm forward flexed at 90°, and the forearm fully supinated. The fulcrum of the goniometer was positioned over the lateral epicondyle of the humerus, with one arm of the device along the length of the humerus to the tip of the acromion process and the other arm along the length of the radius to the radial styloid process [19]. (Figs. 4, 5 and 6)

For ROM measurements, the variables were analyzed continuously.

### Assessment of ultrasonographic findings

Three orthopedic surgeons performed ultrasonography to detect elbow and shoulder abnormalities. Images were obtained using a Philips HDI1 5000 scanner or Philips EPIQ 5 scanner with a variable high-frequency linear-array transducer (7.5 to 10 MHz). Ultrasonographic structural abnormalities over elbow include osteochondritis dissecans of the capitellum when there is a localized flattening of the subchondral bone and a normal outline of the articular cartilage [20], calcification, effusion, avulsion, and partial tear on elbow UCL. Each abnormality was recorded after the surgeon’s confirmation, and the decision was made by consensus.

### Statistical analysis

All data were analyzed using SPSS 25.0 (SPSS Inc., Chicago, Illinois, USA). Epidemiologic data were reported with descriptive statistics, including number, means, standard deviations, range, and percentages where appropriate. Ultrasonographic findings were documented and analyzed using dichotomous categories of either positive or negative findings. ROM measurements of both the shoulder and elbow were documented continuously. For univariate analyses, the *F* test or *t* test was applied for continuous variables, and the chi-square test or Fisher’s exact test was applied for categorical variables. Parameters that were consistently associated with each perspective in the univariate analyses were included in the multivariate analysis. A stepwise multivariate logistic regression analysis was used to identify factors that were related to each clinical finding, with results reported as odds ratios (ORs) and 95% confidence intervals (CI). For all analyses, P values < 0.05 were considered to represent statistical significance.

## Results

The total numbers for each domain may vary. This variation is due to the inability to successfully retrieve certain variables for reasons such as difficulty in recall by the players or inability to cooperate with physical or ultrasonographic examinations.

### Patient demographics

A total of 533 players were included in this study. Among these players, 212 (39.8%) played primary position as pitchers, while 222 (41.7%) played as pitchers and fielders. The overall mean age was 16.4 ± 0.91 years, mean length of official baseball experience was 7.3 ± 2.54 years, mean height was 173.8 ± 6.00 cm, and mean BMI was 23.24 ± 2.913 kg/m^2^ (Table 1a and  1b).

**Table 1a:** Demographic data of the enrolled players

	N	Min	Max	Average	SD	Median
Age (years)	533	13	19	16	0.91	15
Official baseball experience (played years)	533	1	9	7	2.54	5
Height (cm)	533	158	192	173	6.00	
BMI (kg/m^2^)	533	17.4	35.1	23.2	2.913	

**Table 1b:** Demographic data of the players’ dominant hands and defensive positions

	N	Percentage
Laterality		
Left-handed	66	12.4
Right-handed	461	86.5
Switch	6	1.2
Primary position split		
1B	15	2.8
2B	22	4.1
3B	15	2.8
C	48	9.0
CF	11	2.1
IF	98	18.4
LF	11	2.1
OF	71	13.3
P	212	39.8
RF	17	3.2
SS	13	2.4

The demographic data showed that pitchers had greater body height (175.8 ± 6.05 cm vs. 172.4 ± 5.55 cm, *P* < 0.001) and greater elbow total arm angle (135.6 ± 9.27 ° vs. 132.7 ± 9.41 °, *P* < 0.001) (Table 2).

**Table 2:** Analysis grouped by defensive position as pitchers

	Pitchers			Non-pitchers								
	n	Mean	SD	n	Mean	SD	*P*	Min	Max	95% Confidence level		
Age (years)	222	16.5	0.9	310	16.4	0.9	0.17	13	19	-0.047	0.267	
Official baseball experience (years)	222	7.5	2.5	310	7.2	2.5	0.21	1	9	-0.161	0.715	
Started age (years)	221	8.96	2.5	310	9.1	2.4	0.417	7	14	-0.605	0.251	
**Height (cm)	223	175.8	6.1	310	172.4	5.6	< 0.001	158	192	2.422	4.409	
BMI (kg/m^2^)	223	23.0	2.7	310	23.4	3.0	0.07	17.4	35.1	-0.947	0.040	
Shoulder total rotation angle (^o^)	221	159.0	27.0	299	162.0	25.7	0.195	135	185	-7.596	1.554	
Shoulder ER (^o^)	221	104.8	30.3	299	105.7	28.5	0.74	65	100	-6.031	4.274	
Shoulder IR (^o^)	221	54.1	25.3	299	56.3	27.1	0.36	55	105	-6.734	2.448	
**Elbow total arm angle (^o^)	196	135.6	9.3	278	132.7	9.4	< 0.001	110	145	1.217	4.643	

We further grouped the players into pitchers and fielders when analyzing elbow structural abnormalities.

### Elbow ultrasonographic abnormalities

In our study, any type of ultrasonographic structural abnormality in the elbow was recorded as a positive finding for our outcome. As for elbow structural abnormalities, several significant findings were noticed in the study. Longer official baseball experience (*P* < 0.001), younger starting age (*P* < 0.001), less shoulder rotation (*P* = 0.01 and *P* = 0.02 for IR) and lower elbow arm angle (*P* = 0.03) were linked to elbow abnormalities in all players (Table 3). There was no significant correlation noticed concerning fielders (Table 4a), while for pitchers, longer playing experience, early starting age, reduced shoulder rotation and IR, and elbow arm angle were related to elbow structural abnormalities (*P* < 0.001, *P* < 0.001, *P* = 0.01, *P* = 0.05, *P* = 0.03 respectively, Table 4b). Variables that were significantly related to elbow structural abnormalities in each group were further evaluated via multivariate analyses, and the risks were estimated.

**Table 3:** Analysis of elbow structural abnormalities of all players (pitchers and fielders)

	Elbow structural abnormalities +			Elbow structural abnormalities -							
	n	Mean	SD	n	Mean	SD	*P*	Min	Max	95% Confidence level	
Age (years)	126	16.4	1.0	251	16.5	0.9	0.33	13	19	-0.305	0.103
** Official baseball experience (years)	126	7.4	2.5	251	6.6	2.2	< 0.001	1	9	0.306	1.329
** Started age (years)	125	8.9	2.3	251	9.8	2.1	< 0.001	7	14	-1.418	-0.452
Height (cm)	127	173.9	6.2	251	173.7	5.9	0.79	158	192	-1.136	1.494
BMI (kg/m^2^)	127	23.0	2.9	251	23.3	3.0	0.49	17.4	35.1	-0.848	0.407
** Shoulder total rotation angle (^o^)	126	155.1	27.8	242	164.1	29.3	0.01	135	185	-15.25	-2.800
Shoulder ER (^o^)	126	100.4	33.4	240	102.3	32.1	0.59	65	100	-9.105	5.163
** Shoulder IR (^o^)	126	54.7	27.9	240	61.8	29.2	0.02	55	105	-13.230	-0.951
** Elbow total arm angle (^o^)	114	134.3	8.5	212	136.4	8.2	0.03	110	145	-4.001	-0.154

**Table 4a:** Analysis of elbow structural abnormalities of the fielders

	Elbow structural abnormalities +			Elbow structural abnormalities -							
	n	Mean	SD	n	Mean	SD	*P*	Min	Max	95% Confidence level	
Age (years)	67	16.2	0.9	142	16.4	0.9	0.11	13	18	-0.478	0.051
Official baseball experience (years)	67	7.1	2.7	142	6.5	2.2	0.12	1	7	-0.159	1.319
Started age (years)	67	9.1	2.6	142	9.9	2.1	0.30	9	14	-1.513	-0.074
Height (cm)	67	172.5	5.6	142	172.2	5.8	0.67	158	182	-1.306	2.020
BMI (kg/m^2^)	67	22.8	3.2	142	23.5	3.2	0.13	17.4	34.2	-1.645	0.209
Shoulder total rotation angle (^o^)	66	161.0	25.2	132	166.1	30.3	0.21	155	180	-13.600	3.411
Shoulder ER (^o^)	66	104.1	33.4	132	103.1	32.3	0.84	80	100	-8.850	10.839
Shoulder IR (^o^)	66	57.0	32.5	132	63.1	29.2	0.20	60	105	-15.568	3.204
Elbow total arm angle (^o^)	58	135.0	7.77	123	135.8	7.0	0.50	110	145	-3.201	1.569

**Table 4b:** Analysis of elbow structural abnormalities of the pitchers

	Elbow structural abnormalities +				Elbow structural abnormalities -														
	n	Mean		SD		n	Mean		SD		*P*		Min		Max		95% Confidence level		
Age (years)	59	16.5		1.0		109	16.5		1.0		0.89		13		19		-0.295	0.339	
** Official baseball experience (years)	59	7.8		2.2		109	6.7		2.3		< 0.001		3		9		0.369	1.776	
** Started age (years)	58	8.7		1.9		109	9.8		2.9		< 0.001		7		14		-1.729	-0.450	
Height (cm)	60	175.4		6.6		109	175.7		5.5		0.76		158		192		-2.288	1.673	
BMI (kg/m^2^)	60	23.3		2.6		109	22.9		2.8		0.36		17.4		35.1		-0.450	1.219	
** Shoulder total rotation angle (^o^)	60	148.5		29.2		108	161.6		28.1		0.01		135		185		-22.133	-4.002	
Shoulder ER (^o^)	60	96.3		33.2		108	101.4		32.0		0.33		65		100		-15.577	5.296	
Shoulder IR (^o^)	60	52.3		21.7		108	60.2		29.3		0.05		55		105		-15.776	-0.079	
** Elbow total arm angle (^o^)	56	133.6		9.2		89	137.2		9.5		0.03		110		145		-6.746	-0.426	

A number of continuous variables presented an increased risk of elbow structural abnormalities in multivariate analysis. For these measures, the OR indicates that every 1 unit of change (e.g., 1° in ROM) possesses an increased risk of elbow structural abnormalities. Three different models were established for all players: pooled, pitchers, and fielders. When all players were pooled, significant risk factors included started playing baseball at an younger age (OR = 1.202; 95% CI = 1.064–1.357; *P* = 0.003), longer experience of official baseball (OR = 1.154; 95% CI = 1.038–1.283; *P* = 0.008), lower total shoulder rotation angle (OR = 1.007; 95% CI = 1.000–1.014; *P* = 0.050), and less total elbow arm angle (OR = 1.052; 95% CI = 1.017–1.088; *P* = 0.003) (Table 5). For pitchers, significant risk factors included longer experience of official baseball (OR = 1.342; 95% CI = 1.098–1.640; *P* = 0.004), lower total shoulder rotation angle (OR = 1.016; 95% CI = 1.004–1.027; *P* = 0.006), and lower total elbow arm angle (OR = 1.075; 95% CI = 1.024–1.129; *P* = 0.004) (Table 5). There were no significant risk factors for elbow structural abnormalities in fielders.

**Table 5:** Multivariate analysis of elbow structural abnormalities

	OR			95% CI			Max			Min			*P*			
All players pooled																
** Official baseball experience (years)	1.154	1.038			1.283			9			1			0.008		
** Younger age at starting (years)	1.202	1.064			1.357			7			14			0.003		
** Lower total shoulder rotation angle (^o^)	1.007	1.000			1.014			185			110			0.050		
** Lower total elbow arm angle (^o^)	1.052	1.017			1.088			150			110			0.003		
Pitchers																
** Official baseball experience (years)	1.342	1.098			1.640			9			3			0.004		
** Lower total shoulder rotation angle (^o^)	1.016	1.004			1.027			185			135			0.006		
** Lower total elbow arm angle (^o^)	1.075	1.024			1.129			145			110			0.004		

## Discussion

In this study, we analyzed demographic, shoulder and elbow ROM, and ultrasonographic data of high school baseball players in Taiwan. First, the demographic data demonstrated that players with a taller body height and greater elbow ROM were significantly related to pitchers. According to the literature, there is a strong relationship between stride length and ball velocity. The increase in stride length would increase ball velocity, and the average stride length was associated with the body height of pitchers [21, 22]. A study analyzing Major League Baseball (MLB) players between 1985 and 2002, and demonstrated a significant correlation between body height and becoming specifically an established MLB starting pitcher [23]. This finding may denote the preference of coaches for selecting taller players to be pitchers. As for greater elbow ROM, the momentum and spin of the baseball were largely generated in the late cocking, acceleration, and deceleration phases of throwing [24]. As a result, with greater elbow ROM, the thrower could generate a higher spin rate and velocity on the pitch, which would be advantageous for being a pitcher [25]. 

Several studies have addressed the relationship between ROM and elbow injuries in baseball players [12, 19, 26]. In this study, we chose ultrasonographic structural abnormalities of players’ elbows as our grouping standards. In our analysis of elbow structural abnormalities, younger starting ages and more experience in official baseball were significant risk factors for pooled players and pitchers, respectively. Despite the recommendation that young pitcher should participate in sports other than baseball, and should avoid throwing too many pitches during training or competitions [27, 28], some young pitchers today are subjected to early sports specialization and increased training loading in order to meet extreme performance demands [29]. According to a descriptive epidemiology study of 2006–2016, elbow pathology was becoming more prevalent and the mean age of elbow injured players was decreasing [30]. Moreover, greater pitch volume and excessive competition experience without adequate rest gave rise to overuse injuries [31]. In a cross-sectional study of 2019 youth baseball competition in the USA, noncompliance with Pitch Smart guidelines from MLB, which was a regulation concerning training programs and pitch volume restriction designed for young baseball players [32], occurred in more than 90% of teams and almost half of all pitchers [33]. In our study, players that started to play baseball younger and pitchers that were recruited in official baseball trainings for a longer time were prone to have elbow ultrasonographic structural abnormalities, which may be a precursor of elbow injures [34], demonstrating the severity of overuse injuries in youth baseball players.

Repetitive stress of pitching leads to excessive shear forces on the medial aspect of the olecranon tip and olecranon fossa, lateral radio-capitellar compression, posterior extension overload, and medial tension at the UCL [35–37]. These mechanisms causing valgus stress overload may lead to elbow structural abnormalities such as osteophyte formation, loose bodies from fragmentation, and laxity of the UCL, which may give rise to decreased elbow ROM [38, 39]. Several studies have highlighted the difference in ROM between the dominant and non-dominant elbow, with the dominant side possessing decreased ROM [40]. A study revealed that passive range of motion of the throwing elbow significantly decreased shortly after pitching activity, which may be a result of eccentric muscle contractions contributing to acute musculotendinous adaptations [12]. In our study, lower total elbow ROM was a significant risk factor for pooled players and pitchers.

Decreased shoulder ROM with elbow injuries has been reported in several studies [19, 41, 42]. Glenohumeral internal rotation deficit (GIRD) has been regarded as an important risk factor for shoulder injuries in throwing athletes [43–45]. In a retrospective study conducted by Dines et al., there was a positive correlation between GIRD and UCL insufficiency [46]. Moreover, some recent studies revealed that decreased shoulder total range of motion (TROM) was also related to elbow injuries [26]. There have been lots of proposed reasons for this adaptation of TROM decrease on throwing shoulders, including osseous adaptation [45, 47, 48], muscular tightness [49], scapular position, and capsular restriction [50]. Wilk et al., who first introduced the shoulder total rotation concept, in which the amount of shoulder ER and IR at 90° of abduction are added together, emphasized that a decreased total rotation as a risk factor for shoulder injury in several of their studies [18, 51]. In our study, the univariate analysis did demonstrate that a lower shoulder IR was significantly related to elbow structural abnormalities in pitchers and in all players being pooled, which correlated the results being reported in several previous articles [52]. However, shoulder IR was not a significant risk factor in our multivariate model. Particularly, in both univariate and multivariate analyses, in shoulders a decreased total rotational angle was significantly related to elbow structural abnormalities on ultrasonography, which echoes the results of several recent studies [26]. 

Although our study did not provide a definite diagnosis of injury, we found some relationship between decreased shoulder/elbow ROM and elbow structural abnormalities in ultrasonography. Ultrasonographic findings of abnormalities have been regarded as pre-injury status among youth baseball players in several studies [5, 15, 34]. Other diagnostic tools, such as MRI, which require players to spend a great amount of time going to the hospital to undergo the examination, are difficult to apply for young players as a routine checkup because it is time-consuming and expensive. Conversely, regular checkups for shoulder and elbow ROM and ultrasonography are more feasible for most players to prevent early injuries. We believe that the results of our study provide evidence to examine young players in a simple way by not only physicians and surgeons, but also coaches and players.

Our study has several limitations. First, this study was retrospective; therefore, we could only confirm the correlation instead of a precise causal relationship between the recognized risk factors and elbow structural abnormalities. Second, physical and ultrasonographic examinations were not performed on the same day among players, so these players may have been in different phases of the season, which may have influenced the results. Additionally, the shoulder physical examinations were conducted in non-functional positions. Compared to the players’ functional positions, their ROM might be influenced accordingly. Lastly, there was no follow-up on the status of these players, so we may not have confirmed the final injury status of the players with structural abnormalities.

## Conclusion

This study found that our enrolled pitchers had taller body height and greater elbow arm angle. Longer experience of official baseball attendance, lower total rotation angle of shoulders, and lower total arm angle in elbows, especially in the subgroup of pitchers, had an increased risk of ultrasonographic elbow structural abnormalities.

## Data Availability

The details of the data can be found in the Chang Gung Memorial Hospital Database. The point of contact is the corresponding author (Joe Chih-Hao Chiu MD, PhD).

## References

[1] Valovich McLeod TC, Decoster LC, Loud KJ, Micheli LJ, Parker JT, Sandrey MA, White C (2011). National Athletic Trainers’ Association position statement: prevention of pediatric overuse injuries. J Athl Train.

[2] Davis JT, Limpisvasti O, Fluhme D, Mohr KJ, Yocum LA, Elattrache NS, Jobe FW (2009). The effect of pitching biomechanics on the upper extremity in youth and adolescent baseball pitchers. Am J Sports Med.

[3] Matsuura T, Suzue N, Kashiwaguchi S, Arisawa K, Yasui N (2013). Elbow injuries in Youth Baseball players without Prior Elbow Pain: A 1-Year prospective study. Orthop J Sports Med.

[4] Fleisig GS, Weber A, Hassell N, Andrews JR (2009). Prevention of elbow injuries in youth baseball pitchers. Curr Sports Med Rep.

[5] Sakata J, Miyazaki T, Akeda M, Yamazaki T (2021). Return-to-play outcomes in high school baseball players after ulnar collateral ligament injuries: dynamic contributions of flexor digitorum superficialis function. J Shoulder Elb Surg.

[6] Carr JB 2nd, Camp CL, Dines JS. Elbow ulnar collateral ligament injuries: indications, management, and outcomes. Arthroscopy. 2020;36(5):1221–2. 10.1016/j.arthro.2020.02.022 32112818

[7] Vidal VR, Chang MO, Muentes SAG, Ávila AGG, Avila SAG (2022). Ultrasound exploratory study of injuries in baseball athletes: physiotherapy intervention. Linguistics Cult Rev.

[8] Chumbley EM, O’Connor FG, Nirschl RP (2000). Evaluation of overuse elbow injuries. Am Fam Physician.

[9] Patel RM, Lynch TS, Amin NH, Gryzlo S, Schickendantz M. Elbow injuries in the Throwing Athlete. JBJS Rev 2014, 2(11). 10.2106/JBJS.RVW.N.00011 27490404

[10] Conte S, Camp CL, Dines JS (2016). Injury trends in Major League Baseball Over 18 Seasons: 1998–2015. Am J Orthop (Belle Mead NJ).

[11] Tanaka H, Hayashi T, Inui H, Muto T, Ninomiya H, Nakamura Y, Yoshiya S, Nobuhara K (2018). Estimation of shoulder behavior from the viewpoint of minimized shoulder joint load among adolescent baseball pitchers. Am J Sports Med.

[12] Reinold MM, Wilk KE, Macrina LC, Sheheane C, Dun S, Fleisig GS, Crenshaw K, Andrews JR (2008). Changes in shoulder and Elbow Passive Range of Motion after pitching in Professional Baseball players. Am J Sports Med.

[13] Khalil LS, Jildeh TR, Taylor KA, Gulledge CM, Smith DG, Sandberg ML, Makhni EC, Okoroha KR, Moutzouros V (2021). The relationship between shoulder range of motion and elbow stress in college pitchers. J Shoulder Elb Surg.

[14] Pozzi F, Plummer HA, Shanley E, Thigpen CA, Bauer C, Wilson ML, Michener LA (2020). Preseason shoulder range of motion screening and in-season risk of shoulder and elbow injuries in overhead athletes: systematic review and meta-analysis. Br J Sports Med.

[15] Harada M, Takahara M, Sasaki J, Mura N, Ito T, Ogino T (2006). Using sonography for the early detection of elbow injuries among young baseball players. AJR Am J Roentgenol.

[16] Wang Y-L, Chang H-Y, Cheng S-C, Liu C (2016). The effect of age on elbow range of motion in pitchers. Phys Ther Sport.

[17] Garcia GH, Gowd AK, Cabarcas BC, Liu JN, Meyer JR, White GM, Romeo AA, Verma NN (2019). Magnetic resonance imaging findings of the asymptomatic elbow predict injuries and surgery in Major League Baseball pitchers. Orthop J Sports Med.

[18] Manske R, Wilk KE, Davies G, Ellenbecker T, Reinold M (2013). Glenohumeral motion deficits: friend or foe?. Int J Sports Phys Ther.

[19] Garrison JC, Cole MA, Conway JE, Macko MJ, Thigpen C, Shanley E (2012). Shoulder range of motion deficits in baseball players with an ulnar collateral ligament tear. Am J Sports Med.

[20] Takahara M, Shundo M, Kondo M, Suzuki K, Nambu T, Ogino T (1998). Early detection of osteochondritis dissecans of the capitellum in young baseball players. Report of three cases. JBJS.

[21] Manzi JE, Dowling B, Dines JS, Wang Z, Kunze KN, Thacher R, McElheny KL, Carr JB (2021). The association of stride length to ball velocity and elbow varus torque in professional pitchers. J Sports Sci.

[22] Yanagisawa O, Taniguchi H (2020). Relationship between stride length and maximal ball velocity in collegiate baseball pitchers. J Phys Ther Sci.

[23] Greenberg GP. Does a Pitcher’s Height Matter? Baseball Research Journal, (2010).

[24] Fleisig GS, Escamilla RF (1996). Biomechanics of the elbow in the throwing athlete. Oper Tech Sports Med.

[25] Wong R, Laudner K, Evans D, Miller L, Blank T, Meister K (2021). Relationships between clinically measured Upper-Extremity physical characteristics and ball spin rate in Professional Baseball pitchers. J Strength Cond Res.

[26] Wilk KE, Macrina LC, Fleisig GS, Aune KT, Porterfield RA, Harker P, Evans TJ, Andrews JR (2014). Deficits in glenohumeral passive range of motion increase risk of elbow injury in professional baseball pitchers: a prospective study. Am J Sports Med.

[27] Jayanthi N, Pinkham C, Dugas L, Patrick B, Labella C (2013). Sports specialization in young athletes: evidence-based recommendations. Sports Health.

[28] LaPrade RF, Agel J, Baker J, Brenner JS, Cordasco FA, Côté J, Engebretsen L, Feeley BT, Gould D, Hainline B (2016). AOSSM Early Sport specialization Consensus Statement. Orthop J Sports Med.

[29] DiFiori JP, Benjamin HJ, Brenner JS, Gregory A, Jayanthi N, Landry GL, Luke A (2014). Overuse injuries and burnout in youth sports: a position statement from the American Medical Society for Sports Medicine. Br J Sports Med.

[30] Trofa DP, Obana KK, Swindell HW, Shiu B, Noticewala MS, Popkin CA, Ahmad CS (2019). Increasing Burden of Youth Baseball Elbow Injuries in US Emergency Departments. Orthop J Sports Med.

[31] Matsuura T, Iwame T, Suzue N, Arisawa K, Sairyo K (2017). Risk factors for shoulder and elbow pain in youth baseball players. Phys Sportsmed.

[32] Matsuura T, Takata Y, Iwame T, Iwase J, Yokoyama K, Takao S, Nishio S, Arisawa K, Sairyo K (2021). Limiting the Pitch Count in Youth Baseball pitchers decreases Elbow Pain. Orthop J Sports Med.

[33] Greiner JJ, Trotter CA, Walczak BE, Hetzel SJ, Baer GS (2021). Pitching behaviors in Youth Baseball: comparison with the Pitch Smart guidelines. Orthop J Sports Med.

[34] Tajika T, Kobayashi T, Yamamoto A, Kaneko T, Shitara H, Shimoyama D, Iizuka Y, Okamura K, Yonemoto Y, Warita T (2016). A clinical and ultrasonographic study of risk factors for elbow injury in young baseball players. J Orthop Surg (Hong Kong).

[35] Paulino FE, Villacis DC, Ahmad CS (2016). Valgus Extension Overload in Baseball players. Am J Orthop (Belle Mead NJ).

[36] Park JY, Yoo HY, Chung SW, Lee SJ, Kim NR, Ki SY, Oh KS (2016). Valgus extension overload syndrome in adolescent baseball players: clinical characteristics and surgical outcomes. J Shoulder Elb Surg.

[37] Ahmad CS, Conway JE (2011). Elbow arthroscopy: valgus extension overload. Instr Course Lect.

[38] Reddy AS, Kvitne RS, Yocum LA, Elattrache NS, Glousman RE, Jobe FW (2000). Arthroscopy of the elbow: a long-term clinical review. Arthroscopy.

[39] Andrews JR, Craven WM (1991). Lesions of the posterior compartment of the elbow. Clin Sports Med.

[40] Wright RW, Steger-May K, Wasserlauf BL, O’Neal ME, Weinberg BW, Paletta GA (2006). Elbow range of motion in professional baseball pitchers. Am J Sports Med.

[41] Harada M, Takahara M, Mura N, Sasaki J, Ito T, Ogino T (2010). Risk factors for elbow injuries among young baseball players. J Shoulder Elb Surg.

[42] Rosen M, Meijer K, Tucker S, Wilcox CL, Plummer HA, Andrews JR, Ostrander RV (2022). 3rd: shoulder range of motion deficits in Youth throwers presenting with Elbow Pain. Sports Health.

[43] Braun S, Kokmeyer D, Millett PJ (2009). Shoulder injuries in the throwing athlete. J Bone Joint Surg Am.

[44] Cools AM, Johansson FR, Borms D, Maenhout A (2015). Prevention of shoulder injuries in overhead athletes: a science-based approach. Braz J Phys Ther.

[45] Polster JM, Bullen J, Obuchowski NA, Bryan JA, Soloff L, Schickendantz MS (2013). Relationship between Humeral Torsion and Injury in Professional Baseball pitchers. Am J Sports Med.

[46] Dines JS, Frank JB, Akerman M, Yocum LA (2009). Glenohumeral internal rotation deficits in baseball players with ulnar collateral ligament insufficiency. Am J Sports Med.

[47] Chant CB, Litchfield R, Griffin S, Thain LM (2007). Humeral head retroversion in competitive baseball players and its relationship to glenohumeral rotation range of motion. J Orthop Sports Phys Ther.

[48] Crockett HC, Gross LB, Wilk KE, Schwartz ML, Reed J, O’Mara J, Reilly MT, Dugas JR, Meister K, Lyman S (2002). Osseous adaptation and range of motion at the glenohumeral joint in professional baseball pitchers. Am J Sports Med.

[49] Posner M, Cameron KL, Wolf JM, Belmont PJ, Owens BD (2011). Epidemiology of Major League Baseball injuries. Am J Sports Med.

[50] Reagan KM, Meister K, Horodyski MB, Werner DW, Carruthers C, Wilk K (2002). Humeral retroversion and its relationship to glenohumeral rotation in the shoulder of college baseball players. Am J Sports Med.

[51] Wilk KE, Meister K, Andrews JR (2002). Current concepts in the rehabilitation of the overhead throwing athlete. Am J Sports Med.

[52] Fleisig GS, Bolt B, Fortenbaugh D, Wilk KE, Andrews JR (2011). Biomechanical comparison of baseball pitching and long-toss: implications for training and rehabilitation. J Orthop Sports Phys Ther.

